# AnnotaPipeline: An integrated tool to annotate eukaryotic proteins using multi-omics data

**DOI:** 10.3389/fgene.2022.1020100

**Published:** 2022-11-22

**Authors:** Guilherme Augusto Maia, Vilmar Benetti Filho, Eric Kazuo Kawagoe, Tatiany Aparecida Teixeira Soratto, Renato Simões Moreira, Edmundo Carlos Grisard, Glauber Wagner

**Affiliations:** ^1^ Laboratório de Bioinformática, Universidade Federal de Santa Catarina (UFSC), Campus João David Ferreira Lima, Florianópolis, Brazil; ^2^ Instituto Federal de Santa Catarina (IFSC), Campus Lages, Lages, Brazil; ^3^ Laboratório de Protozoologia, Universidade Federal de Santa Catarina (UFSC), Campus João David Ferreira Lima, Florianópolis, Brazil

**Keywords:** workflow, proteogenomics, genome annotation, functional annotation, hypothetical proteins

## Abstract

Assignment of gene function has been a crucial, laborious, and time-consuming step in genomics. Due to a variety of sequencing platforms that generates increasing amounts of data, manual annotation is no longer feasible. Thus, the need for an integrated, automated pipeline allowing the use of experimental data towards validation of *in silico* prediction of gene function is of utmost relevance. Here, we present a computational workflow named AnnotaPipeline that integrates distinct software and data types on a proteogenomic approach to annotate and validate predicted features in genomic sequences. Based on FASTA (i) nucleotide or (ii) protein sequences or (iii) structural annotation files (GFF3), users can input FASTQ RNA-seq data, MS/MS data from mzXML or similar formats, as the pipeline uses both transcriptomic and proteomic information to corroborate annotations and validate gene prediction, providing transcription and expression evidence for functional annotation. Reannotation of the available *Arabidopsis thaliana*, *Caenorhabditis elegans, Candida albicans, Trypanosoma cruzi,* and *Trypanosoma rangeli* genomes was performed using the AnnotaPipeline, resulting in a higher proportion of annotated proteins and a reduced proportion of hypothetical proteins when compared to the annotations publicly available for these organisms. AnnotaPipeline is a Unix-based pipeline developed using Python and is available at: https://github.com/bioinformatics-ufsc/AnnotaPipeline.

## Introduction

Genome annotation involves a detailed description and understanding of the genome structure and assignment of biological functions to the genes (Stein, 2001). Structural annotation thus characterizes the physical structure of coding and non-coding regions on a given genome, resulting in a physical map of the genes’ number and positioning. Along determination of the structure and organization of the protein-coding sequences (CDS) located within open reading frames (ORF) of each gene, annotation also includes a description of other genomic elements such as promoters and enhancers (Korf, 2004; Danchin et al., 2018). Several computational tools known as gene predictors, such as AUGUSTUS (Stanke and Waack, 2003) and GeneMark (Brůna, Lomsadze, and Borodovsky, 2020), have been widely used to perform structural annotation (Yandell and Ence, 2012).

Functional annotation consists of assigning biological information to genes, such as their involvement in biological processes, molecular functions, presence of functional protein domains, and subcellular localization, among others (Stein, 2001; Yandell and Ence, 2012). The assignment of biological functions to protein-coding genes is generally performed through similarity analysis with databases containing previously annotated protein sequences using sequence aligners such as BLAST (Camacho et al., 2009) or DIAMOND (Buchfink, Reuter, and Drost, 2021). The biological function of a predicted CDS is therefore assumed to be the same as the protein in the database that demonstrates the most significant similarity, leading to an annotation transfer (Hegyi and Gerstein, 2001). Thus, the accuracy of the annotated database is fundamental for genome annotation, allowing the quality of downstream analyses based on the transferred annotations. Especially with the use of high-throughput sequencing during the past years, several public genomic and proteomic databases from a variety of organisms are nowadays available. However, the exponential growth of datasets impairs the quality of a proper and detailed structural and functional annotation of genomes. For that, the use of curated databases such as SwissProt/UniProtKB (The UniProt Consortium, 2021) and Ensembl (Flicek et al., 2014), or even organism-specific databases, such as those contained in the VEuPathDB (Amos et al., 2022), is highly recommended to ensure high quality to the genome annotation.

Considering the growing datasets of genomic and proteomic databases, and the specific genomic features across taxa, combining different computational tools or pipelines to automatically assess gene structural and functional annotation has been widely used (Danchin et al., 2018). Composed of a set of data processing methods connecting inputs and outputs in series, automated pipelines can perform genome annotation by sequence similarity (Hyatt et al., 2010; Steinbiss et al., 2016) or functional annotation of proteins (Gotz et al., 2008; Vlasova et al., 2021; Törönen and Holm, 2022). Nevertheless, only a few genome annotation pipelines use expression experimental data (RNA-Seq or MS/MS) to validate the *in silico* annotation (Ghali et al., 2014; Sheynkman et al., 2014).

Large-scale genomic and transcriptomic studies based on high-throughput sequencing platforms in the past decade have provided increasing amounts of data (Kumar et al., 2016a), also providing extensive gene expression profiles based on transcribed RNAs (RNA-seq) sequencing. Moreover, extensive proteomic data acquired from sensitive mass spectrometry (MS) technologies are available from several databases (Vaudel et al., 2016), such as PRIDE (Perez-Riverol et al., 2022), MassIVE (Miao et al., 2012), and the ProteomeXchange Consortium (Vizcaíno et al., 2014). Thus, using transcription and expression evidence to annotate newly predicted CDS or reannotate formerly analyzed genomes would reveal novel biological aspects. The proteogenomic approach allows the cross-validation of genomic, transcriptomic, and proteomic data on both intra- and inter-specific analyzes (Nesvizhskii, 2014). However, this approach requires novel computational methods and pipelines. Thus, integrating the classic annotation analysis by sequence similarity with customizable parameters and databases, combined with functional prediction validated with RNA-seq and MS/MS data evidence, would enhance genome annotation as an essential step toward comprehending biological mechanisms.

In this study, we developed AnnotaPipeline, a proteogenomic computational tool for automatic annotation of eukaryotic genomes using support from high-throughput transcriptomic and proteomic data, allowing validation of gene function and expression.

## Methods

### AnnotaPipeline

#### Development and overview

**FIGURE 1:**
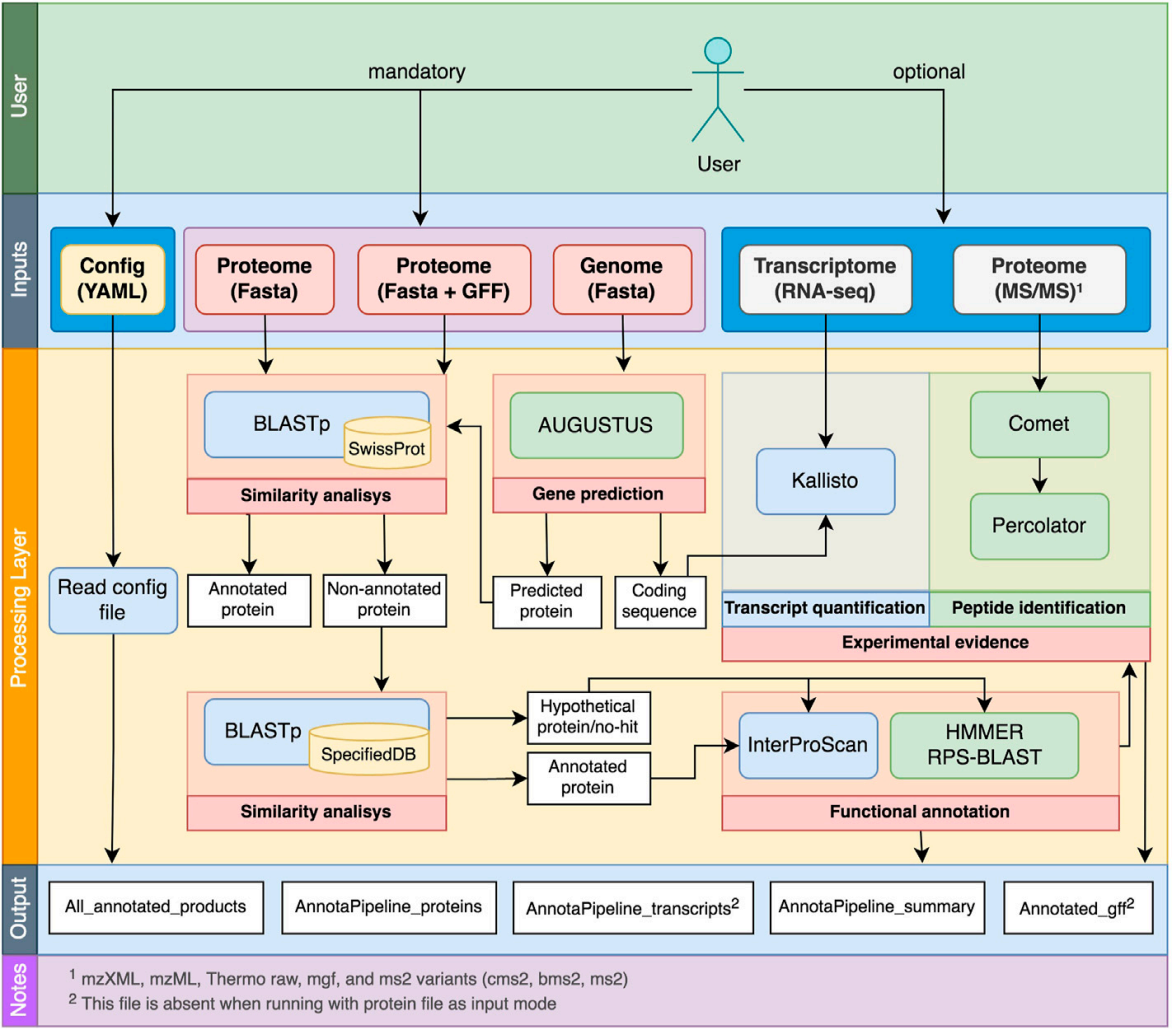
Overview of AnnotaPipeline workflow, indicating the optional and the required inputs from the user, the internal processes, and the output layers.

The AnnotaPipeline overall scheme and processes are shown in Figure 1. This pipeline was developed using Python and runs on Unix-based systems, consisting of a series of tolls and in-house scripts for data preparation, processing, and analysis. Documentation related to installation instructions and scripts to run AnnotaPipeline are available at https://github.com/bioinformatics-ufsc/AnnotaPipeline.

#### Input and configuration files

AnnotaPipeline requires the input of at least one of the following different FASTA files: 1) a nucleotide sequence file, 2) a protein sequence file, 3) a protein sequence file, and structural annotation files in GFF3 format. If the first option is selected, AnnotaPipeline will perform gene prediction on the provided nucleotide sequence. Therefore, it is essential to use a trained AUGUSTUS model for the gene prediction process before executing the pipeline. This execution will produce an annotated GFF3, and CDS sequences will contain a complete header. For the second option, gene prediction will be skipped, and the final output file will contain only a simplified sequence header. The third option is executed equally to the second option, the pipeline will include annotations for each CDS from the provided GFF file. Also, it is recommended that the submitted GFF file is in GFF3 format, preferably from a previous AUGUSTUS gene prediction.

Aside from the molecular data input, it is also required from the user to access the YAML configuration file prior to running the pipeline, where locations of both software and databases required for the personalized analysis must be provided. Similarly, if analyses with experimental data will be carried out, it is also necessary to provide the locations of folders containing RNA-seq and MS/MS data.

Users can define the number of processing threads that will be used during the execution of the pipeline (default is set to 4 threads) and are required to define the cutoff parameters and specific keywords to classify hypothetical proteins during the similarity analysis process. This configuration step is facilitated if the user installs AnnotaPipeline using Conda from the environment file available at https://github.com/bioinformatics-ufsc/AnnotaPipeline.

#### Annotation process

The annotation process starting with a genomic file input is divided into three steps. Initially, gene prediction is performed by AUGUSTUS (Stanke and Waack, 2003). Although AnnotaPipeline is mainly focused on eukaryotic organisms, the pipeline accepts input of further gene prediction training models if absent in the AUGUSTUS standalone version. It is recommended to use the WebAUGUSTUS platform to generate custom training models (Hoff and Stanke, 2013).

Following gene prediction, the annotation process continues into similarity analysis performed by the BLASTp algorithm (Camacho et al., 2009) using (i) the SwissProt database, which contains about 570,000 manually curated protein sequences from a wide variety of organisms (The UniProt Consortium, 2021), and (ii) a user-specified database such as TrEMBL/UniProtKB, VEuPathDB and GenBank NR, or additional databases that must be specified in the AnnotaPipeline.yaml configuration file. Despite the used database, the pipeline contains parsing scripts that automatically will transfer the protein annotation for the predicted CDS on the output file. Proteins are then classified into three groups: annotated proteins (known function), hypothetical proteins, and no-hit proteins. Annotated proteins are those with attributed annotation either by the SwissProt or the user-specified database. In AnnotaPipeline, hypothetical proteins are considered those presenting similarities with proteins with no specific annotation in the databases (unknown function) and that contain filter keywords in their descriptions, such as “fragment”, “hypothetical”, “partial”, “uncharacterized”, “unknown”, and “unspecified”. These are the default keywords used by the pipeline, but users can change these in the AnnotaPipeline.yaml configuration file. Annotations in subject proteins will be disregarded if at least one description contains any of the provided keywords. No-hit proteins are proteins with no available match, and therefore no annotation, in either database used in the similarity analysis step. For downstream analysis steps, the no-hit and the hypothetical proteins are grouped by the pipeline. Furthermore, proteins revealing no matches with databases and presenting no supporting evidence from experimental data are considered true negative proteins.

The third step consists of the functional annotation of proteins, starting with analyzing both annotated and hypothetical protein groups by InterProScan software (Jones et al., 2014). Exclusively for the hypothetical protein/no-hit group, further analysis using the hmmscan algorithm of the HMMER suite (Finn, Clements, and Eddy, 2011) and the RPS-BLAST (Camacho et al., 2009) are performed. The resulting functional annotation is contained in a single output file where all predicted proteins will be annotated and can be used as input for the experimental validation analyses.

### Experimental validation with proteogenomic data

The AnnotaPipeline accepts the input of RNA-seq and MS/MS data that will allow experimental validation of CDS prediction and annotation. Upon activation of the experimental analysis module, transcriptomic data will be processed by Kallisto (Bray et al., 2016), which performs a pseudo-alignment of RNA-seq reads to the annotated protein file. The result will be refined based on a quantification of aligned transcripts, which are accounted for transcripts per million (TPM). Users may concatenate their transcriptomic data into a single FASTQ file (for single-end RNA-seq) or two FASTQ files (R1 and R2, for paired-end RNA-seq) to run multiple experiments at once. For experimental validation using proteomic data (MS/MS), users can provide a single folder containing their MS/MS data files to run multiple experiments simultaneously. The search for MS/MS-derived peptides among the annotated proteins will be performed using Comet (Eng, Jahan, and Hoopmann, 2013), following the user-provided search parameters in comet.params configuration file, generating the input for the Percolator software (The et al., 2016). Then, the proteomic data will be searched among the annotated proteins dataset and parsed by the q-value threshold of the Percolator software.

#### Output files

The pipeline will create a log file and an output folder in the AnnotaPipeline directory. The log file contains details of script processing, software execution, and outputs of each computational tool. Also, this log may contain any possible warnings or errors relative to the software execution. Within the output folder, the pipeline will create (i) two FASTA files containing the annotated proteins and their respective annotated CDS, (ii) a GFF file including a transcript product field containing the final annotation for each CDS, (iii) a TXT file containing the all CDS product ID and annotated description, and (iv) a TSV file summarizing all annotated CDS and information regarding transcription (RNA-Seq) or expression (MS/MS) evidence. In addition to these main output files, within each of the folders created by AnnotaPipeline, other outputs can help the user manually curate the annotations suggested by the pipeline (Supplementary Table S1).

#### Comparative evaluation of AnnotaPipeline performance

Performance tests were carried out using a computational cluster equipped with 40 threads processor (3.2 GHz), 285 GB RAM memory (DDR4, 2,400 MHz), and 5 TB storage space (2.5 SATA HD, 7,200 RPM). Storage was mainly used for RNA-seq and MS/MS data of the testing organisms. Despite the availability of computing power, the number of processing threads used for testing was set to 12 in the AnnotaPipeline.yaml configuration file.

Molecular data from three different model organisms were used to test AnnotaPipeline: *Arabidopsis thaliana* (strain TAIR10), an essential model for plant biology and genetics; *Caenorhabditis elegans* (strain WBcel235), an important model for molecular and developmental biology; and *Candida albicans* (strain SC5314), a fungal pathogen model. Genomic data for each of these organisms were retrieved from GenBank under the following accession numbers: GCA_000001735.2, GCA_000002985.3, and GCA_000182965.3, respectively. RNA-seq data for each of these organisms were obtained from BioProject/NCBI under the following accession numbers: PRJNA779571, PRJNA809747, and PRJNA750749 for *A. thaliana*; PRJNA734346, PRJNA658149, and PRJNA755869 for *C. elegans*; PRJNA714869, PRJNA496318, PRJNA752883, and PRJNA744166 for *C. albicans*. MS/MS data for each of these organisms were obtained from ProteomeXchange, under the following accession numbers: PXD012708 and PXD010730 for *A. thaliana*; PXD025128 for *C. elegans*; PXD005364 for *C. albicans*.

For the similarity analysis step, in addition to the SwissProt database, a specific database of protein sequences was used for each model organism: for *A. thaliana*, a subset of 370,680 protein sequences was obtained from the GenBank NR dataset; for *C. albicans*, the FungiDB v56 containing 2,331,868 protein sequences was obtained from VEuPathDB; and for *C. elegans*, a subset of 23,010 protein sequences was obtained from TrEMBL.

AnnotaPipeline was independently run with default parameters for every organism, using the genome FASTA file obtained for each organism as input. AUGUSTUS (version 3.4.0) prediction was performed with the gene model argument set to partial and using the prediction model dataset already provided by the software, as in: arabidopsis, for *A. thaliana*; *candida*_albicans, for *C. albicans*; and *caenorhabditis*, for *C. elegans*. Therefore, the gene prediction step was not optimized. BLASTp (version 2.12.0) execution was done assuming an e-value of 1e-5, the number of maximum target sequences set to 10. Also, a minimum threshold value of sequence coverage was set to 30, sequence identity 40, and sequence positivity 60 for the annotation transfer. The annotation was chosen based on the highest bit score between the analyzed sequences.

InterProScan (version 5.52–86.0) was run for the functional annotation step, allowing for the lookup of corresponding Gene Ontology annotation (--goterms). HMMscan (version 3.3.2) had the e-value of both sequences and domains set to 1e-5, and RPSblast (version 2.12.0) also had the minimum e-value of target sequences set to 1e-5. Kallisto (version 0.48.0) pseudo-alignment of RNA-seq dataset was run with 1,000 bootstraps, and the minimum threshold of TPM was selected as the mean. Comet (version 2021.01) was run for each MS/MS dataset with a scan range minimum and a maximum set to 200 and 4,000, respectively. After, Percolator (version 3.5) was run with Comet output files, and the results obtained were filtered by a q-value threshold of 0.05. As a complete example, all the output files from the *A. thaliana* dataset are available at https://github.com/bioinformatics-ufsc/AnnotaPipeline/blob/v1.0/Output%20Example/Annota_Athaliana.tar.xz.

The pipeline was further tested using two taxonomically close protozoa species of medical relevance containing over 50% of their CDS annotated as hypothetical proteins: *Trypanosoma cruzi* (strain Sylvio X10/1), the etiological agent of Chagas disease (Talavera-López et al., 2021) and *Trypanosoma rangeli* (strain SC58) an avirulent trypanosomatid of mammals (Stoco et al., 2014). Genomic data was retrieved from the TriTrypDB (version 57) under the following accession numbers: DS_107bdce9bb, and DS_9d0531db8e, respectively. For both organisms, the Augustus prediction model was trained online based on their respective available genome file and annotated transcripts files (tcruzi_sylviox10, for *T. cruzi*; and trypanosoma_rangeli, for *T. rangeli*). For the similarity analysis step, a database of 648,560 protein sequences obtained from the TriTrypDB was used, along with the mandatory SwissProt database. The AnnotaPipeline was run using default parameters for both trypanosomatid species, as previously mentioned.

## Results

### AnnotaPipeline workflow

The complete execution of AnnotaPipeline resulted in the expected output files that were named <basename>_AnnotaPipeline_<file>.<format>, allowing users to identify the results and perform multiple experiments in the same directory by swapping the <basename> of the experiments in the AnnotaPipeline.yaml configuration file.

The generated annotation files in FASTA format display for each sequence a header containing the following information separated by a pipe character “|”: sequence identification; source organism; scaffold number; CDS start; CDS end; strand orientation; and sequence description, were functional annotations provided by GO and IPR are included. If no structural annotation GFF file is included in the analysis, information concerning strand orientation and scaffold location will be absent. Also, AnnotaPipeline changes the “transcript product” field of each CDS in the annotated GFF file to the corresponding sequence description present in the header of the FASTA file.

### Comparative analysis of AnnotaPipeline results

AnnotaPipeline was comparatively tested using genomic data of different model organisms for which genome annotation is available. The pipeline enabled experimental evidence analyses and no gene prediction optimization. The summary of the obtained annotations, functional annotations, and experimental evidence results for the *A. thaliana*, *C. albicans*, and *C. elegans* datasets are presented in Table 1.

**TABLE 1:** Summary of AnnotaPipeline annotations, functional annotations, and experimental evidence results for different model organisms.

Parameter	*Arabidopsis thaliana* TAIR10		*Candida albicans* SC5314		*Caenorhabditis elegans* WBcel235	
	GenBank	AnnotaPipeline	GenBank	AnnotaPipeline	GenBank	AnnotaPipeline
Predicted proteins	27,562	19,651	6,043	5,377	19,984	14,278
Annotated proteins	25,151 (91.25%)	19,434 (98.90%)	3,735 (61.81%)	5,349 (99.48%)	13,186 (65.98%)	10,587 (74.15%)
Annotated by SwissProt	–	13,444 (69.18% of annotated)	–	2,914 (54.48% of annotated)	–	5,395 (50.96% of annotated)
Annotated by SpecificDB	–	5,990 (30.82% of annotated)	–	2,435 (45.52% of annotated)	–	5,192 (49.04% of annotated)
Hypothetical proteins	2,411	169	2,308	13	6,798	3,229
No hit proteins (true negative)*	–	48 (45)	–	15 (9)	–	462 (440)
Total hypothetical proteins	2,411 (8.75%)	217 (1.10%)	2,308 (38.19%)	28 (0.52%)	6,798 (34.02%)	3,691 (25.85%)
Proteins with at least 1 IPR term	–	17,974 (91.47%)	–	4,704 (87.48%)	–	11,050 (77.39%)
Proteins with at least 1 GO term	–	13,612 (69.27%)	–	3,705 (68.90%)	–	7,587 (53.14%)
Proteins with transcript evidence	–	3,228 (16.43%)	–	716 (13.32%)	–	1,714 (12.0%)
Proteins with peptide evidence	–	1,546 (7.87%)	–	809 (15.05%)	–	0 (%)

*True negative are proteins with no match on studied databases and no supporting evidence from experimental data, which could possibly be artifacts from gene prediction. Reference genome GenBank accession number: *Arabidopsis thaliana* (strain TAIR10) = GCA_000001735.2; *Caenorhabditis elegans* (strain WBcel235) = GCA_000002985.3; *Candida albicans* (strain SC5314) = GCA_000182965.3.

For *A. thaliana*, the pipeline annotated a total of 19,651 protein sequences in 29 h and 07 min; 5,377 protein sequences for *C. albicans* in 10 h and 06 min; and 14,278 protein sequences for *C. elegans* in 20 h and 58 min.

Among the genome analyzed, *C. albicans* had the highest percentage of annotated proteins with 99.48%, followed by *A. thaliana* with 98.90%. *C. elegans* had 22.62% of their protein sequences annotated as hypothetical proteins, and another 3.24% of proteins with no matches available in the analyzed databases. Comparatively to the current data from analyzed genomes available in public databases, AnnotaPipeline provided a higher number of annotated proteins (known function) and fewer hypothetical proteins. Consequently, the number of hypothetical proteins in the *A. thaliana* dataset went down from 8.75% to 1.10% using the AnnotaPipeline, while for *C. elegans* and *C. albicans* datasets, the reduction was from 34.02% to 25.85% and 38.19%–0.52%, respectively.

Functional annotation of the *A. thaliana*, *C. albicans* and *C. elegans* genomes using the AnnotaPipeline revealed 69.27%, 68.90%, and 53.14% of their CDS associated with at least one GO term associated, respectively. When RNA-Seq and MS/MS data were included for the analysis of experimental evidence of transcription or expression, *A. thaliana*, *C. albicans* and *C. elegans* had 16.43%, 15.05%, and 12.00% of their annotated proteins validated with transcriptomic and proteomic data, respectively. Interestingly, no *C. elegans* annotated CDS were validated by the available MS/MS dataset.

Comparative analysis of the genome annotation for *T. cruzi* and *T. rangeli* retrieved from the TriTrypDB (version 57) and the annotation generated using AnnotaPipeline is shown in Supplementary Table S2. Although not including experimental data for validation (RNA-Seq or MS/MS), the pipeline was able to reduce the number of hypothetical proteins by 60.46% and 42.84% for *T. cruzi* and *T. rangeli*, respectively, while increasing the proportion of annotated CDS having at least one GO term assigned (Supplementary Table S2).

Considering the annotation provided by AnnotaPipeline, it is possible to classify the annotated protein sequences into eight different categories based on three different criteria: 1) available annotation based on sequence similarity with provided databases; 2) transcription evidence by quantifying RNA-seq reads; and 3) translation evidence supported by the identification of peptides matches from MS/MS information. As an example, result of the analysis of the *A. thaliana* dataset is shown in Table 2. From a total of 19,651 annotated CDS, the less represented categories are those who contains CDS having support from either RNA-Seq (12.65%) or MS/MS (4.09%) support, or both (3.78%).

**TABLE 2:** Classification table of annotated proteins by AnnotaPipeline for the *Arabidopsis thaliana* dataset.

Categories	Hypothetic Annotation	Transcript Evidence	Peptide Evidence	Number of sequences	Percentage (%)
1	Yes	No	No	203	1.03
2	No	No	No	15,417	78.45
3	Yes	Yes	No	5	0.03
4	Yes	No	Yes	7	0.04
5	No	Yes	No	2,480	12.62
6	No	No	Yes	796	4.05
7	Yes	Yes	Yes	2	0.01
8	No	Yes	Yes	741	3.77

## Discussion

Whole genome annotation is one of the first and most essential steps in any genome study, consisting in a time-consuming and laborious work depending on the genome size, and no longer can be performed manually due to the amount of data generated by high-throughput sequencing (Ouzounis and Karp, 2002). AnnotaPipeline was designed to perform automatic annotation of genomes, having the unique feature to include experimental data derived from transcriptomic (RNA-Seq) or proteomic (MS/MS) approaches towards experimental validation of an annotated CDS. The pipeline is easy to install, runs on operating systems that support command-line options, such as Unix-based systems, and does not require high computational demands, although the time-consuming tasks can be reduced while using more robust machines. It is also user-friendly and customizable to meet the user needs in terms of analysis stringency.

Although distinct genome annotation pipelines are available (Gotz et al., 2008; Hyatt et al., 2010; Ghali et al., 2014), AnnotaPipeline provides the possibility of using RNA-seq and MS/MS data to improve genome annotation simultaneously. Considering that proteomic data have become increasingly accessible (Nesvizhskii, 2014), and new RNA-seq technologies, such as single-cell or single-molecule sequencing, are improving significantly (Wang et al., 2019), the use of this pipeline would increase que quality and accuracy of the annotated genomes from a variety of organisms by providing several possible annotations for each protein sequence. On top of providing a more accurate automated analysis, the pipeline also offers information to support manual curation of the annotation by the user.

Comparison of the results obtained using AnnotaPipeline with the data available in public databases, it was possible to observe a reduction in the number of hypothetical proteins for *A. thaliana* (91.0%), *C. elegans* (45.70%), and *C. albicans* (98.79%), as shown in Table 1. This reduction can be due to the use of customizable databases and keywords but also to the use of combined proteogenomic data to complement gene annotation, increasing the reliability of gene prediction and automatic annotation.

In addition to these well-annotated genomes, AnnotaPipeline also showed good performance when used to annotate the repetitive genomes from two closely related species of *Trypanosoma* (*T. cruzi* and *T. rangeli*) retrieved from TriTrypDB, both lacking RNA-seq or MS/MS data for experimental validation. It was possible to observe a relative reduction of more than 60% in the number of proteins annotated as hypothetical (Supplementary Table S2).

The use of experimental data to validate CDS annotation raises a critical discussion, especially regarding hypothetical proteins. Categorizing hypothetical proteins according to their evidence of transcription or expression by AnnotaPipeline revealed interesting results. Although presenting experimental support from RNA-Seq, MS/MS or both, as observed for *A. thaliana* proteins belonging to Class 7 (Table 2), they remain annotated as hypothetical proteins in the studied databases. In this context, annotation pipelines using this multi-omics approach can provide fundamental insights into new and uncharacterized proteins and revise those whose functions are already annotated. Knowledge areas associated with medicine would benefit most since previously annotated hypothetical proteins could now be studied and thus allow for the re-evaluation of disease diagnosis or prognostic methods (Kumar et al., 2016b).

Furthermore, AnnotaPipeline can be used to guide the exploration of proteins because it adds functional annotation to protein annotation through the incorporation of GO and IPR terms. Especially for hypothetical or uncharacterized proteins, the classical description of annotations might not be biologically informative, so the lack of functional annotations (such as GO or IPR terms) increases this information gap (Lubec et al., 2005; Gotz et al., 2008). AnnotaPipeline provides descriptive and functional information for these proteins during the automated annotation process, which helps to identify potential prediction artifacts and streamline the process of manually curating the annotations. Lastly, the AnnotaPipeline summary file can provide to users the SUPERFAMILY protein information, adding yet another layer of detail to annotations. This information can provide new insights into the functionality of uncharacterized proteins, as they represent possibilities of new structures and functions to be explored (Lubec et al., 2005).

## Conclusion

By integrating experimental data from RNA-seq and MS/MS analyses to validate prediction and annotations of protein-coding sequences, AnnotaPipeline, an integrated and modular genomic annotation pipeline, promoted the reduction of the number of hypothetical proteins for various organisms. The use of this original proteogenomic approach on reannotation of *A. thaliana*, *C. elegans*, *C. albicans*, *T. cruzi*, and *T. rangeli* datasets, have increased the proportion of annotated proteins, consequently reducing the number of hypothetical proteins if compared to the currently available annotation. AnnotaPipeline was developed as a generalist annotation pipeline, allowing the assessment of genomes from any eukaryotic organism with available molecular data.

## Data Availability

The datasets presented in this study can be found in online repositories. The names of the repository/repositories and accession number(s) can be found in the article/Supplementary Material.
