# Supplementary material for: AnnotaPipeline: An integrated tool to annotate eukaryotic proteins using multi-omics data
Source: Front Genet. 2022 Nov 22;13:1020100. doi: 10.3389/fgene.2022.1020100 (PMC9723129; doi:10.3389/fgene.2022.1020100)
Supplement: Supplementary file 1 [file Table2.docx]

**Supplementary Table 2.** Comparative analysis of the TriTrypDB and AnnotaPipeline annotations of *Trypanosoma cruzi* and *Trypanosoma rangeli* genomes.

| **Parameter** | ***Trypanosoma cruzi***  **Sylvio X10/1** | | ***Trypanosoma rangeli***  **SC58** | |
| --- | --- | --- | --- | --- |
|  | **TriTrypDB** | **AnnotaPipeline** | **TriTrypDB** | **AnnotaPipeline** |
| Predicted proteins | 20,619 | 9,127 | 7,475 | 5,649 |
| Annotated proteins | 5,075 (24.61%) | 7,759 (85.01%) | 2,400 (32.11%) | 4,234 (74.95%) |
| Annotated by  SwissProt | - | 2,569 (33.11% of annotated) | - | 1,252 (29.57% of annotated) |
| Annotated by  SpecificDB | - | 5,190 (66.89% of annotated) | - | 2,982 (70.43% of annotated) |
| Hypothetical  Proteins | 15,544 | 1,348 | 5,075 | 1,411 |
| No hit proteins | - | 20 | - | 4 |
| Total hypothetical  proteins | 15,544 (75.39%) | 1368 (14.99%) | 5,075 (67.89%) | 1,415 (25.05%) |
| Proteins with at  least 1 IPR term | - | 5,757 (63.08%) | - | 4,052 (71.73%) |
| Proteins with at  least 1 GO term | - | 3,809 (41.73%) | - | 2,990 (52.93%) |

Reference genome TriTrypDB (version 57) accession number: *Trypanosoma cruzi* (strain Sylvio X10/1) = DS_107bdce9bb; *Trypanosoma rangeli* (strain SC58) = DS_9d0531db8e.
